# Successful Control of Methicillin-Resistant *Staphylococcus aureus* in Endemic Neonatal Intensive Care Units—A 7-Year Campaign

**DOI:** 10.1371/journal.pone.0023001

**Published:** 2011-08-12

**Authors:** Yhu-Chering Huang, Rey-In Lien, Lin-Hui Su, Yi-Hong Chou, Tzou-Yien Lin

**Affiliations:** 1 Division of Pediatric Infectious Diseases, Chang Gung Memorial Hospital at Linkou, Kweishan, Taoyuan, Taiwan; 2 Division of Neonatology, Chang Gung Memorial Hospital at Linkou, Kweishan, Taoyuan, Taiwan; 3 Committee of Infection Control, Chang Gung Memorial Hospital at Linkou, Kweishan, Taoyuan, Taiwan; 4 Department of Laboratory Medicine, Chang Gung Memorial Hospital at Linkou, Kweishan, Taoyuan, Taiwan; 5 School of Medicine, Chang Gung University, Kweishan, Taoyuan, Taiwan; Baylor College of Medicine, United States of America

## Abstract

**Background:**

Methicillin-resistant *Staphylococcus aureus* (MRSA) is among the most important nosocomial pathogens in the intensive care unit (ICU) worldwide, including Taiwan. Since 1997, our neonatal ICUs (NICUs) had become endemic for MRSA.

**Methodology/Principal Findings:**

To control MRSA spread in our NICUs, we implemented a series of infection control measures stepwise, including reinforcement of hand hygiene since January 2000, augmentation of aseptic care over the insertion site of central venous catheter since July 2001, introduction of alcohol-based handrubs since April 2003, surveillance culture for MRSA and cohort care for the colonized patients between March 2003 and February 2004, and surveillance culture with subsequent decolonization of MRSA between August 2005 and July 2006. After implementation of these measures, MRSA healthcare-associated infection (HAI) density reduced by 92%, from 5.47 episodes per 1000 patient-days in 1999 to 0.45 episodes per 1000 patient-days in 2006; MRSA bloodstream infection reduced from 40 cases in 1999 to only one case in 2006. Compared to those obtained during the period of surveillance culture without decolonization, both rates of MRSA colonization (8.6% vs. 41%, *p*<0.001) and infection (1.1% vs. 12%, *p*<0.001) decreased significantly during the period of surveillance and decolonization. Molecular analysis of the clinical isolates during the study period showed that the endemic clone, which dominated between 1998 and 2005, almost disappeared in 2006, while the community clones increased significantly in 2006–2007.

**Conclusion/Significance:**

Through infection control measures, MRSA HAIs can be successfully controlled, even in areas with high levels of endemic MRSA infections such as our NICUs.

## Introduction

Methicillin-resistant *Staphylococcus aureus* (MRSA) is among the most important pathogens of bacteremia in the intensive care units (ICU). Nowadays, MRSA becomes endemic in most hospitals around the world [1] [2] and accounts for 40–60% of all healthcare-associated *S. aureus* infections. Colonized patients are the major reservoirs of MRSA in hospitals. Colonizing strains may serve as endogenous sources for overt clinical infections or may spread to other patients [3]–[11]. To reduce and control healthcare-associated infections (HAIs) caused by MRSA, a “search and destroy” strategy, which first detects the patients with MRSA colonization and then decolonizes the MRSA with certain antimicrobial agents, was recently proposed and implemented in some hospitals of different countries, with inconsistent effects [12]–[16].

In Taiwan, MRSA was first documented in early 1980s and rapidly increased in 1990s [17]. In 2000, methicillin resistance had been identified in 53–83% of all *S. aureus* isolates in 12 major hospitals of Taiwan [18]. In our neonatal ICUs (NICUs), *S. aureus* is the leading pathogen of HAIs and MRSA represented majority of all the *S. aureus* isolates since 1997. Between 1997 and 1999, the prevalence of *S. aureus* among the HAIs increased significantly from 32.4% in 1997, 43.6% in 1998, to 52.6% in 1999. The percentage of MRSA among *S. aureus* isolates also rose significantly from 87.2% in 1997, 92.1% in 1998, to 95.1% in 1999 [19]. Apparently, our NICUs were endemic for MRSA. We then implemented a series of infection control interventions stepwise in our NICUs to try to reduce HAIs caused by MRSA. After a 7-year campaign, MRSA HAIs was successfully controlled temporarily with the implementation of the strategy of “search and destroy”. Here, we report our experiences for MRSA control in the NICUs.

## Methods

Chang Gung Children's Hospital is a university-affiliated teaching hospital, situated in northern Taiwan, that provides a range of care, from primary to tertiary care, and is a part of Chang Gung Memorial Hospital (CGMH). There are three NICUs, distributed on 2 floors, in this children's Hospital. Currently, there are 17 and 20 beds in NICU-1, and NICU-2, respectively. NICU-3 included two areas, 12 level -III beds in area 1 and 45 non-level-III beds in area 2 (special care nurseries). All the healthcare-associated infections (HAIs) in three NICUs from 1999 to 2007 were prospectively collected and recorded according to the standard definition of HAIs [20].

### Ethics

The study included the institution's healthcare infection data, which were routinely collected and reported by the institution's infection control committee, and was also among the institution's quality-improvement programs proposed by the institution's infection control committee. Since active surveillance for MRSA control is considered to be quality improvement, IRB approval was not required to be included when application for the grants and thus the study was not reviewed by the institutional review board (IRB) of Chang Gung Memorial Hospital at that time and informed consent could be waived [21].

### Implementation of infection control interventions

Since 2000, a series of infection control interventions were implemented stepwise in our NICUs to try to reduce healthcare-associated infections caused by MRSA (Table 1). We firstly re-enforced hand washing before and after contact with the infants hospitalized in NICUs since January 2000 by increasing infection control education of, increasing infection control practitioner's audits of, and feedback of HAIs data to the health care workers (HCWs) working in NICUs. From a case-control study conducted in 2001, we found that the presence of skin infection at onset was one of the risk factors for MRSA bacteremia in these infants [22]. Standardized operation procedures for the insertion and the continuous care of peripherally inserted central venous catheter (PICC) were revised, aiming to accelerate the placement process (by a designated team) and to improve the aseptic care over the insertion site. Briefly, after successful insertion, 10% povidine-iodine containing alcohol (75%) was applied to the insertion site, normal saline used to decolorize, and the area was covered by a transparent dressing (“Tegaderm”). Nurses checked the insertion site frequently and changed the dressing every 3 days. The PICC lines were not impregnated with antibacterial or antiseptic agents and antibiotic lock prophylaxis was not used. The strategy commenced in July 2001.

**Table 1:** Infection control measures implemented in the neonatal intensive care units of Chang Gung Children's Hospital between 1999 and 2007.

Interventions	Period implemented
Augmenting hand washing before and after contact with patients	Jan 2000∼present
Revision of standardized operation procedures for the insertion and continuous care of PICC	Jul 2001∼present
Institution of alcohol-based handrubs	Apr 2003∼present
Surveillance culture for MRSA carriage and cohort care	Mar 2003∼Feb 2004
Surveillance culture for MRSA carriage and intranasal application of mupirocin ointment	Aug 2005∼Jul 2006

PICC, peripherally inserted central venous catheter; MRSA, methicillin-resistant *Staphylococcus aureus*.

From March 2003 to February 2004, screening for MRSA carriage among the hospitalized infants at NICU-1 and -2 was conducted [23], which was supported by the research grants. During the NICUs stay, specimens from the nares, postauricular areas, axillae, and umbilicus were obtained weekly and sent for detection of MRSA. The infants with MRSA colonization, if identified, were separated from non-colonized infants and placed in a segregated area of the units, and cohort care by designated nurses was implemented. Almost at he same time, the outbreak of severe acute respiratory syndrome (SARS) occurred in Taiwan, and alcohol-based handrubs were introduced into the hospital in April 2003 and were used in these NICUs thereafter.

From August 2005 to July 2006, we implemented the “search and destroy” strategy into NICU-1 and -2, which was also supported by the research grants. From the previous surveillance study [22], we learned that nearly 90% of the colonized infants are detected within the first 2 weeks of admission and sampling of both nares and umbilicus is adequate for surveillance cultures in this population. Hence, during this period, only specimens from both nares and umbilicus were obtained, within 24 hours of admission and then weekly for two weeks (3 times in total). In addition to placing the colonizing infants in a segregated area and cohort care, decolonization procedures with topical mupirocin ointment application to nares and umbilical area were administered twice daily for five consecutive days if they still stayed in the NICUs. If an infant with MRSA colonization had MRSA clinical isolates, the clinical isolates as well as the colonized isolates were genotyped and compared. MRSA isolates recovered from clinical diagnostic samples (beyond surveillance culture specimens) submitted to the clinical microbiologic laboratory were regarded as clinical isolates. In accordance with the standard definition of HAIs [20], any infant with clinical isolates of MRSA who was receiving antimicrobial therapy was categorized as experiencing an episode of infection. However, from August 2006 to October 2007, no active surveillance for MRSA was conducted in these NICUs since no research grants supported.

Surveillance cultures for health care workers (HCWs) were performed, during surveillance periods, and specimens were obtained from the nares of HCWs working in both units. Intranasal mupirocin treatment was applied to the nares of each HCW with MRSA colonization.

### Collection, selection and genotyping analysis of MRSA isolates

Specimens for surveillance culture were obtained with a cotton swab, placed in a transport medium (Venturi Transystem), and then processed in the microbiology laboratory within 4 hours. Identification of MRSA was confirmed according to National Committee for Clinical Laboratory Standards guidelines [24].

Except for year 2002, MRSA clinical isolates from the hospitalized infants at these NICUs between 1998 and 2007 were collected and selected for genotyping analysis. For those years with more than 40 clinical MRSA isolates collected (129 isolates in 2001,166 isolates in 2003, 140 isolates in 2004, 46 isolates in 2005), at least 40 isolates per year were selected for analysis. For years 2006 and 2007, all the clinical isolates were molecularly characterized since less than 40 clinical isolates per year were identified. A total of 429 clinical isolates were selected for molecular analysis. Part of the genotyping results for a substantial number of MRSA isolates from the years 1999, 2000 and 2003 were published previously [23],[25], which are also displayed in this study. Colonized isolates from the infants and HCWs were also molecularly characterized.

The molecular methods included pulsed-field gel electrophoresis (PFGE) with *Sma*I digestion, staphylococcal chromosomal cassette (SCC*mec*) typing, and multilocus sequence type (MLST). In addition, the presence of Panton-Valentine leukocidin (PVL) genes was also examined. All the procedures were described previously [25]–[30]. The genotypes of PFGE were designated, as in our previous studies [25]–[29], in alphabetical order; any new type, if identified, was designated consecutively. PFGE patterns with <4-band differences from an existing genotype were defined as subtypes of that genotype and were labeled with Arabic number suffixes. Two isolates were considered to be indistinguishable, related, or distinct if they had the same subtype, the same genotype, or a different type, respectively.

### Statistic analysis

We compared MRSA colonization and subsequent infection between the infants with and without topical mupirocin traetment by means of χ^2^ (continuity-adjusted) or Student's *t* tests. Relative risk and/or odds ratios (ORs) were calculated with 95% confidence intervals (CIs). Healthcare-associated infection density and MRSA HAI density from 1999 to 2007 were analyzed by Mantel-Haenszel Chi-square test. Statistical analyses of the data were performed with EpiInfo, version 6 (Centers for Disease Control and Prevention, Atlanta, GA) and SAS for Windows, version 6.11 (SAS Institute, Cary, NC).

## Results

### Yearly healthcare-associated infection density

**Figure 1:**
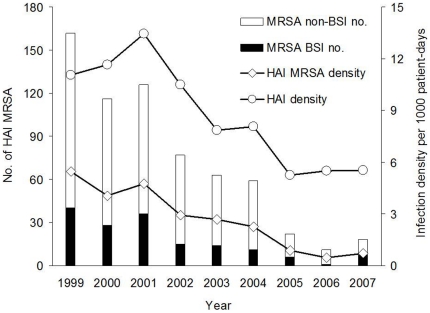
Healthcare-associated infection (HAI) density, overall and caused by methicillin-resistant *Staphylococcus aureus* (MRSA), in the neonatal intensive care units of Chang Gung Children's Hospital significantly decreased from 1999 to 2007. HA MRSA infection episodes, overall and bloodstream, also decreased markedly.

Through these infection control measures, HAIs in these 3 NICUs caused by MRSA as well as by all bacterial pathogens decreased gradually and significantly from 1999 to 2007 (Table 2 and figure 1). The HAI density decreased from 13.5 episodes per 1000 patient-days in 2001 to 5.50 episodes per 1000 patient-days in 2006 (*p*<0.001). Ratio of HAIs caused by MRSA also decreased from 50% in 1999 to 8.3% in 2006 (*p*<0.001) and the HAI bloodstream infection caused by MRSA decreased from 40 cases in 1999 to only one case in 2006. However, all these indices rebounded in 2007 after surveillance culture for MRSA was discontinued.

**Table 2:** Healthcare-associated infections (HAIs) in the neonatal intensive care units of Chang Gung Children's Hospital between 1999 and 2007.

Year	Patient-days	HAI episodes	HAI density* (‰)	HAI by *S. aureus* No. (%)			HA BSI No. (%)		
				*S. aureus*	MRSA	MRSA HAI density* (‰)	All pathogens	*S. aureus*	MRSA
1999	29,609	327	11.04	171(52)	162(50)	5.47	146	43(29)	40(27)
2000	28,517	332	11.64	117(35)	116(35)	4.06	138	28(20)	28(20)
2001	26,452	356	13.46	128(36)	126(35)	4.76	124	36(29)	36(29)
2002	26,335	276	10.48	78(28)	77(28)	2.92	95	15(16)	15(16)
2003	23,658	186	7.86	67(36)	63(34)	2.66	59	17(29)	14(24)
2004	26,108	211	8.08	61(29)	59(28)	2.26	71	12(17)	11(16)
2005	24,866	130	5.23	28(22)	22(17)	0.88	60	8(13)	6(10)
2006	24,199	133	5.50	19(14)	11(8.3)	0.45	56	1(1.8)	1(1.8)
2007	25,284	140	5.54	23(16)	18(13)	0.71	66	9(14)	7(11)

BSI, bloodstream infection; MRSA, methicillin-resistant *Staphylococcus aureus*.

*Healthcare-associated infection (HAI) density and MRSA HAI density significantly decreased from 1999 to 2007 by Mantel-Haenszel Chi-Square test (*p*<0.001) for trend.

### Effect of infection control measures

The reduction of MRSA infection was prominently for two specific time periods, from 2001 to 2002 and from 2004 to 2005. For the first time period, we conducted a revised standardized operation procedure for insertion and continuous care of peripherally inserted central venous catheter (PICC), with augmentation of aseptic procedure and care of the insertion of central venous catheter. For the second time period, we conducted the “search and destroy” strategy.

The results of surveillance culture without decolonization were published previously [22] and are summarized in Table 3. Briefly, MRSA colonization was detected for 41% of 783 infants surveyed during their NICU stay and was noted for 91% of 92 infants with MRSA infections. Previous colonization was detected in 68 episodes (81%) of MRSA infections; colonized and clinical isolates were indistinguishable in 63 episodes (93%).

**Table 3:** Comparison of methicillin-resistant *Staphylococcus aureus* colonization and infection between the infants hospitalized at neonatal intensive care units with and without mupirocin treatment.

Characters	No mupirocin treatmentNo. (%)	Mupirocin treatmentNo. (%)	*p* value	Odds ratio (95% confidence interval)
Period	3/2003–2/2004	8/2005–7/2006		
No. subjects	783	450		
Male	442 (56)	254 (56)	0.999	1 (0.79–1.27)
Prematurity	468 (60)	305 (68)	0.005	0.71 (0.55–0.91)
GA<32 weeks	210 (27)	139 (31)	0.127	0.82 (0.63–1.07)
Birth body weight <2500gms	452 (58)	268 (60)	0.531	0.93 (0.73–1.18)
Birth body weight <1500gms	157 (20)	124 (28)	0.002	0.66 (0.5–0.87)
Inborn	399 (51)	241 (54)	0.379	0.9 (0.71–1.14)
Admission <7 days old	654 (84)	393 (87)	0.072	0.74 (0.52–1.04)
No. infection	92 (12)	5 (1.1)	<0.001	11.85 (4.6–33.3)
No. colonized	323 (41)	39 (8.7)	<0.001	7.4 (5.1–10.76)
With infection	84 (10.7)	1 (0.22)	<0.001	53.96 (8.1–1048)
No. non-colonized	460 (59)	410 (91)	<0.001	0.14 (0.1–0.2)
With infection	8 (1.02)	4 (0.89)	0.819	1.15 (0.31–4.56)
HCW colonized	6/123 (4.9)	5/85 (5.9)	0.764	0.82 (0.21–3.23)

GA, gestational age; prematurity, GA<37 weeks; HCW, health care worker.

During the period of surveillance and decolonization, MRSA colonization was detected for 8.6% of 452 infants surveyed. Of the 39 infants with colonization, intranasal mupirocin ointment was administered to 26 infants who still stayed in the NICUs. Follow-up cultures were obtained from 18 infants and showed positive in two infants. Second course of intranasal mupirocin ointment was administered in both infants and subsequently eradicated MRSA in both cases. One of them developed MRSA sepsis before the second course of therapy was commenced. All 4 isolates (2 clinical and 2 colonized isolates) from this case were genetically indistinguishably. In addition to this case, 4 additional MRSA infected cases were identified, without previous colonization. Five (5.9%) of 85 health care workers were colonized with MRSA. The comparison between the two periods is shown in Table 3 and significant difference was noted in terms of rates of infection, colonization and colonization with subsequent infection. However, no significant difference was noted in terms of rates of MRSA non-colonized but with infection and colonization of health care workers.

### Molecular characteristics of MRSA clinical and colonized isolates

The detailed molecular characteristics of MRSA isolates are shown in Table 4. From the 429 clinical isolates analyzed, a total of 7 pulsotypes were identified. There were two major clones and characterized as sequence type (ST) 239 (or its single locus variant)/pulsotype A (Hungary clone)/SCC*mec* III or IIIA/PVL-negative, accounting for 62% of the isolates, and ST59/pulsotype C/SCC*mec* IV/PVL-negative, accounting for 26%. The former clone was dominant (>50% of the isolates) from 1998 to 2004, became weakened (39% of the isolates) in 2005, reached zero in 2006, and then resurged in 2007 (18% of the isolates). The latter clone remained steady (around 30%) during the study period, except in 1998, 2001 and 2003 (no more than 10%). Two community clones, characterized as ST 59/pulsotype D/SCC*mec* V_T_/PLV-positive and ST 59/pulsotype AN/SCC*mec* IV/PVL-negative, emerged and increased markedly in 2007. The clone of PFGE pattern A significantly decreased while pattern D significantly increased from 1999 to 2007 by Mantel-Haenszel Chi-Square test (*p*<0.001) for trend.

**Table 4:** Molecular characteristics of methicillin-resistant *Staphylococcus aureus* isolates from the hospitalized infants in NICUs between 1998 and 2007.

Category	Year	No. isolates	PFGE patterns§						
			A	C	D	F	H	U	AN
Clinical	1998	10	9(90)	1(10)	0	0	0	0	0
	1999	56	34(61)	22(39)	0	0	0	0	0
	2000	56	34(61)	20(36)	1(1.8)	0	1(1.8)	0	0
	2001	40	37(93)	3(7.5)	0	0	0	0	0
	2003	93	85(91)	3(3.2)	5 (5.4)	0	0	0	0
	2004	69	41(59)	26(38)	2 (2.9)	0	0	0	0
	2005	46	18(39)	20(43)	5(11)	1(2.2)	0	2(4.3)	0
	2006	21	0	7(33)	14(67)	0	0	0	0
	2007	38	7(18)	9(24)	8(21)	1(2.6)	0	2(5.3)	11(29)
	Total	429	265(62)	111(26)	35(8.1)	2(0.47)	1(0.23)	4(0.93)	11(2.6)
Colonized in infants	2003–04	464	418(90)	43(9.3)	3(0.6)	0	0	0	0
	2005–06	61	8(13)	34(56)	15(25)	0	0	0	4(6.6)
Colonized in HCWs	2003–04	30	5(17)	23(77)	0	0	0	0	0
	2005–06	5	0	4(80)	0	0	0	0	1(20)
MLST type			239, 1310*	59	59	5		573#	59
PVL-positive			0/106	1/74	29/29	0/2		0/4	1/11
SCC*mec*			III, IIIA (105/106), UT (1/106)	IV (74/74)	V_T_ (28/29), IV (1/29)	II (2/2)		UT (4/4)	IV (11/11)

PFGE, pulsed-field gel electrophoresis; SCC*mec*, staphylococcal chromosomal cassette; PVL, Panton-Valentine leukocidin; MLST, multilocus sequence type; UT, untypeable.

*single locus variant of ST 239.

#single locus variant of ST 1.

§PFGE pattern A significantly decreased while pattern D significantly increased from 1999 to 2007 by Mantel-Haenszel Chi-Square test (*p*<0.001) for trend.

Among the 525 colonized MRSA isolates from the infants analyzed, four PFGE patterns were identified. Ninety percent of the 464 isolates from the surveillance period (2003–2004) belonged to ST239/pulsotype A, while more than 80% of the 61 colonized isolates from the period of surveillance with decolonization (2005–2006) belonged to the linage of ST59. In contrast, most colonized isolates from HCWs, regardless of during which period, belonged to the clone of ST59/pulsotype C/SCCmec IV/PVL-negative.

## Discussion

The present study demonstrates that through infection control measures, HAIs caused by MRSA can be successfully controlled temporarily, even in high level MRSA endemic neonatal intensive care units. In the current study, MRSA HAI density was reduced by 92%; HA bloodstream infection caused by MRSA was reduced from 40 cases per year to only one case per year. With the reduction of HAI caused by MRSA, the HA infection density decreased proportionally and significantly. It appears that zero HA MRSA bacteremia in NICUs is not infeasible, even in MRSA endemic units like ours, if effective infection control measures are implemented and executed strictly.

During the study period, neither the manpower of nursing staff nor the bed occupation rate (more than 95%) in our NICUs was changed. The reduction of MRSA infection was gradual and significantly; it appeared to occur prominently for two specific time periods, from 2001 to 2002 and from 2004 to 2005. For the first time period, we revised the standardized operation procedure for insertion and continuous care of peripherally inserted central venous catheter (PICC). This strategy was based on our findings from the case-control study that the presence of skin infection at onset was associated with MRSA bacteremia in these infants. Augmentation of aseptic procedure and care of the insertion of central venous catheter (CVC) has been documented to be able to reduce the incidence of catheter-related bacteremia [31] and seemed to be somewhat effective in our NICUs. Conducting the strategy of surveillance culture with subsequent decolonization of MRSA carriage in two of three NICUs was the major change of infection control measures in the second time point in the current study. Compared to those during the period of surveillance culture without decolonization, both MRSA colonization rate and MRSA infection rate among the NICU infants decreased significantly. In contrast, the rate of MRSA infection among the non-colonized infants was similar during both periods; even, MRSA HA infection rate increased slightly in 2007 when the strategy of “search and destroy” was discontinued since the supported grant was due. (Afterwards, another project with a cross-over design was granted and conducted since November 2007) Altogether, these findings seemed to suggest that topical mupirocin treatment may effectively decolonize MRSA carriage in these infants and reduce the subsequent MRSA infection. Though the susceptibility test of MRSA isolates to mupirocin was not performed in this series, we believe that most isolates were susceptible to mupirocin since this medication, though licensed, had not been used in Taiwan for years. However, since no control group (only historical control) was included and not every infant with MRSA colonization received topical mupirocin therapy in the current study, further studies are needed to elucidate this issue [32]–[35].

Compared to adult ICUs, patients admitted to NICUs are relatively “simple”. Most infants hospitalize immediately after birth and a substantial proportion of the infants are inborn. In addition, vertical transmission of MRSA is infrequently seen in newborns. Therefore, most infants do not have MRSA colonization on admission to NICUs. Then, if the HCWs working in and the environmental objects in NICUs are free form MRSA, the hospitalized infants would not acquire MRSA colonization and/or infection during their NICU stay. In the current study, between 2003 and 2006, nasal MRSA carriage rate among HCWs working in our NICUs ranged from 4.8% to 13% for 4 surveys [23]. But no survey for environmental objects was conducted during the study period. However, the condition would not change, even though MRSA may be introduced into the units anytime, if each HCW can perform hand hygiene exactly and strictly. These may partly explain why the “search and destroy” strategy can be effective in our NICUs but not so effective reported from adult ICUs otherwise [14].

From the molecular analysis of MRSA isolates, we found that the epidemic as well as endemic clone, ST239/pulsotype A (Hungary or Brazilian clone), dominated between 1998 and 2005 in our NICUs, even accounting for 90% of the clinical and colonized isolates in certain years. This clone was almost eradicated, not identified from any clinical isolate, from our NICUs in 2006; however, it still accounted for 13% of the colonized isolates during 2005–2006. This clone returned and identified from clinical isolates again in 2007 and persisted in 2008 (data not shown here) but accounted for less than 20% of the clinical isolates. The clone, ST239/pulsotype A, had prevailed in our hospital (CGMH) and even the whole island between 1992 and 2001, accounting for 54% to 93% of clinical isolates from different hospitals islandwide [26],[36]–[39]. However, the predominance was decreasing during 2004–2005 in our hospital (CGMH) [38], so was in our NICUs in the current study almost at the same time. In contrast, the clone of ST59/pulsotype C/SCCmec IV/PVL-negative accounted for one-fourth of the clinical isolates and remained relatively steady throughout the study period. The clone accounted for most colonized MRSA isolates from healthy children [27] as well as a substantial proportion of community-associated MRSA infection in Taiwan [28],[29],[40]–[42] and thus was categorized as a community strain recently. More than 70% of colonized isolates from HCWs working in these NICUs also belonged to this clone during the study period, suggesting that they might acquire MRSA colonization in the community rather than in the hospital. In addition, two other community clones, also belonging to ST59 linage, emerged and increased markedly during 2006–2007. It has been reported that community-associated MRSA strains spread into the hospital and even successfully replaced the original hospital strains [43],[44]. The changing molecular epidemiology needs more surveillance and its clinical implication and significance needs more observations.

Results from the surveillance culture, we reported previously [23], indicated that preceding or concurrent colonization was detected for >80% of the infants with MRSA infection, and the clinical isolates were indistinguishable with the colonized isolates in >90% of the episodes, on the basis of molecular evidence. These strongly suggest the association between MRSA colonization and subsequent infection and indirectly suggest that to reduce MRSA infection in the infants hospitalized in NICUs, active surveillance with subsequent decolonization of MRSA is mandatory. As the issue which disinfectants or antimicrobials, topical or systemic administration, are effective deserve further studies.
